# Innovative Chair and System Designs to Enhance Resistance Training Outcomes for the Elderly

**DOI:** 10.3390/healthcare12191926

**Published:** 2024-09-26

**Authors:** Teng Qi, Miyuki Iwamoto, Dongeun Choi, Siriaraya Panote, Noriaki Kuwahara

**Affiliations:** 1 Doctoral Program of Advanced Fibro-Science, Kyoto Institute of Technology, Kyoto 606-8585, Japan; d3851001@edu.kit.ac.jp; 2 Department of Social System Studies, Doshisha Women’s College of Liberal Arts, Kyoto 610-0395, Japan; m-iwamoto@dwc.doshisha.ac.jp; 3 Department of Informatics, The University of Fukuchiyama, Kyoto 620-0886, Japan; choi-dongeun@fukuchiyama.ac.jp; 4 Faculty of Information and Human Sciences, Kyoto Institute of Technology, Kyoto 606-8585, Japan; spanote@kit.ac.jp

**Keywords:** elderly, resistance training, chair-based exercise, physical stability, movement accuracy, MediaPipe, joint angles

## Abstract

Introduction: This study aims to provide a safe, effective, and sustainable resistance training environment for the elderly by modifying chairs and movement systems used during training, particularly under unsupervised conditions. Materials and Methods: The research focused on investigating the effect of modified chair designs on enhancing physical stability during resistance training by involving 19 elderly participants (mean 72.1, SD 4.7). The study measured changes in the body’s acceleration during movements to compare the effectiveness of the modified chairs with those commonly used in chair-based exercise (CBE) training in maintaining physical stability. A system was developed based on experimental video data, which leverages MediaPipe to analyze the videos and compute joint angles, identifying whether the actions are executed correctly. Results and Conclusions: Comparisons revealed that modified chairs offered better stability during sitting (*p* < 0.001) and stand-up (*p* < 0.001) resistance training. According to the questionnaire survey results, compared to the regular chair without an armrest, the modified chair provided a greater sense of security and a better user experience for the elderly. Video observations indicated that the correct completion rate for most exercises, except stand-up resistance training, was only 59.75%, highlighting the insufficiency of modified chairs alone in ensuring accurate movement execution. Consequently, the introduction of an automatic system to verify proper exercise performance is essential. The model developed in this study for recognizing the correctness of movements achieved an accuracy rate of 97.68%. This study proposes a new chair design that enhances physical stability during resistance training and opens new avenues for utilizing advanced technology to assist the elderly in their training.

## 1. Introduction

As the elderly population grows, there is an escalating demand for medical resources, particularly highlighted by a deficit in healthcare services targeting older adults. According to the United Nations 2023 report, the global population aged 65 and over is projected to increase from 761 million in 2021 to approximately 1.6 billion by 2050 [1]. This demographic transition emphasizes the critical need for maintaining elderly health.

Frailty is a multidimensional and dynamic condition, theoretically defined as “a state of increased vulnerability, resulting from age-associated declines in reserve and function across multiple physiologic systems, such that the ability to cope with every day or acute stressors is compromised” [2]. Frailty is prevalent not only among the elderly but also in adults under 65 years of age. In samples from Sweden and the UK, the pooled prevalence rates were 10.3% (95% CI: 2.7–32.7) for individuals aged ≤ 55, 14.4% (95% CI: 4.5–37.2) for those aged 55–64, and 19.2% (95% CI: 2.5–68.5) for those aged 65–74 [3]. In Japan, the prevalence of frailty is about 5% among those aged 65 to 69 and increases with age, reaching around 35% in individuals over 80 [4]. Although the characteristics of frailty may vary with age, the risk factors are largely similar across both early-life and late-life stages [3]. Frailty is a common and clinically significant condition among older adults, primarily because it is associated with adverse health outcomes, including hospitalization, falls, disability, and mortality [3]. Research indicates that 42% of frail elderly individuals have been hospitalized in the past year, in contrast to 22% of those in a pre-frail state and 11% among non-frail individuals [5]. The prevention of frailty is paramount; it not only enhances the quality of life for seniors but also alleviates the strain on healthcare systems [6,7]. Timely medical access, consistent physical activity, and regular health assessments can delay the onset and progression of frailty, thereby promoting healthier and more sustainable aging trajectories [8].

Sarcopenia is defined as the progressive loss of skeletal muscle mass, strength, and power and is considered a key element of frailty [9]. Therefore, it is essential to implement regular exercise programs focusing on muscle training and balance skills to prevent frailty in the elderly [10]. It is known that muscle mass decreases with age, and the rate of muscle loss varies by body part. Mr Tanimoto measured the muscle mass of 4003 elderly Japanese individuals and obtained trends in muscle mass changes in the upper limbs, trunk, lower limbs, and the entire body [11]. According to Mr Tanimoto’s findings, consistent with the results of Gallagher D, the rate and extent of muscle loss are greatest in the lower limbs [12]. This decline in physical and muscular strength, especially in the lower limbs, heightens the risk of falls and accidents [13]. Therefore, this study particularly focuses on lower limb muscle training.

Resistance training involves repetitive movements that place a load on muscles. The amount of load can be adjusted according to the individual’s condition and goals using their body weight, elastic tubes, dumbbells, or machines [14]. While resistance training using machines is effective even for frail elderly individuals, it requires a trained professional to set up [15]. Compared to machine-based resistance training, resistance training that uses the individual’s body weight maintains or enhances muscular strength and reduces the physical strain on elderly individuals during the training process [16]. Bodyweight resistance training is not only beneficial in preventing sarcopenia but is also among the most suitable preventive resistance training for the elderly to perform [16].

Currently, Japanese government websites and books [17,18] provide programs and introductions to bodyweight resistance training for the elderly, aiming to improve their physical health and quality of life. Japanese researchers have specifically analyzed the effects of lower limb bodyweight resistance training [19]. The results have demonstrated that resistance exercises using the weight of the lower limbs are effective in enhancing muscular strength among the elderly [19].

The barriers reported by elderly individuals participating in resistance training include safety concerns, fear, health-related issues, pain, fatigue, and a lack of social support [20]. To ensure that elderly individuals can safely and beneficially incorporate strength training into their lives, the necessity of evidence-based guidelines and safe equipment for resistance exercises is emphasized [21].

This study is based on the chair-based exercise (CBE) program, integrated with existing resistance training movements, aimed at preventing frailty in the elderly [19,22,23]. CBE primarily employs seated exercises, using chairs to enhance sitting and standing stability, and is considered an essential part of a comprehensive exercise regimen designed specifically for frail older adults [24]. Chair-based exercise (CBE) has been proven effective and should be promoted as a simple and accessible activity to maintain and develop strength in older adults [25].

In this study, the chair used for resistance training was modified to enhance both safety and accuracy during lower limb bodyweight resistance training for the elderly at home, under unsupervised conditions. Additionally, the effectiveness of the modified chair in maintaining the physical stability of the elderly was verified, and the feasibility of using changes in joint angles to assess the accuracy of the movements was demonstrated.

## 2. Materials and Methods

### 2.1. Selection of Movement and Chairs

This study draws on muscle resistance training movements referenced in studies from the UK’s NHS and other research, confirming their effectiveness in maintaining muscular strength among individuals aged 60 and over [19,22,23,26]. Here, nine movements targeting different muscle groups in the legs are detailed as follows:

- M1: Anterior tibialis and peroneal muscles—Toe and heel raises

Key action: Performed sitting or standing, alternately lifting the toes and then the heels to strengthen the muscles on the front and outer sides of the foot.

- M2: Quadriceps—Single-leg raises and knee extension

Key action: Lift one leg while seated, extending the knee. Then, slowly move the leg back and forth to stimulate the quadriceps.

- M3: Tensor fasciae latae, sartorius and gluteus medius—Knee squeezes and leg opening

Key action: Start seated with knees together, then open and close the legs laterally.

- M4: Iliopsoas and abdominal muscles—Knee lifts

Key action: Lift the knees towards the chest while seated to strengthen the iliopsoas and abdominal muscles.

- M5: Stand-up training

Muscle areas: Quadriceps, gluteal muscles, abdominal muscles

Key action: Repeatedly perform the action of standing up from a chair to strengthen the lower body and core muscles.

- M6: Peroneal muscles—Heel raises

Key action: While standing, raise and lower the heels to strengthen the peroneal and other lower leg muscles.

- M7: Quadriceps—Heel raises and knee bend

Key action: While standing, strengthen the quadriceps by raising the heels while bending the knees.

- M8: Gluteus medius—Lateral single-leg raises

Key action: While standing, raise one leg to the side to train the gluteus medius and improve balance.

- M9: Iliopsoas—High knee lifts

Key action: Alternately lift the knees high while standing to strengthen the iliopsoas and improve overall stability.

Armless chairs have been utilized in resistance training studies for the elderly and are also recommended on the NHS website [19,23,26]. In the “Sitting Exercises” section of the NHS website, it is advised that chairs with armrests be avoided as they may restrict the movement of older individuals [26]. In this study, a similarly sized and more commonly available armless chair was selected for use in subsequent research. The specific details of the chair used in this study are as follows: the model CK-S890VR64N, manufactured by KOKUYO, lacks armrests and is produced in Japan. Although these chairs are widely used due to their simple structure, they lack several important features necessary to support the specific training regimes identified in this study.

### 2.2. Chair Design Issues and Improvements

Based on the issues identified previously, specific solutions were developed to address these challenges. Initially, the blueprint of the developed chair was presented, detailing its dimensions and structure. Subsequently, the three main parts of the proposed chair were discussed, with explanations provided for the functional reasons and methods of use for each (Table 1). This approach enables readers to understand how the proposed design contributes to supporting resistance training routines for the elderly, providing a clear insight into the specific mechanisms involved.

#### 2.2.1. Problem 1

Based on problem 1, one of the proposed chair improvements in this study is the addition of a grip on both sides of the seat [29]. This design is intended to help the elderly maintain physical stability during exercise. In particular, the use of these gripping areas during standing and sitting movements will allow the elderly to perform these movements more safely and confidently.

**Figure 1:**
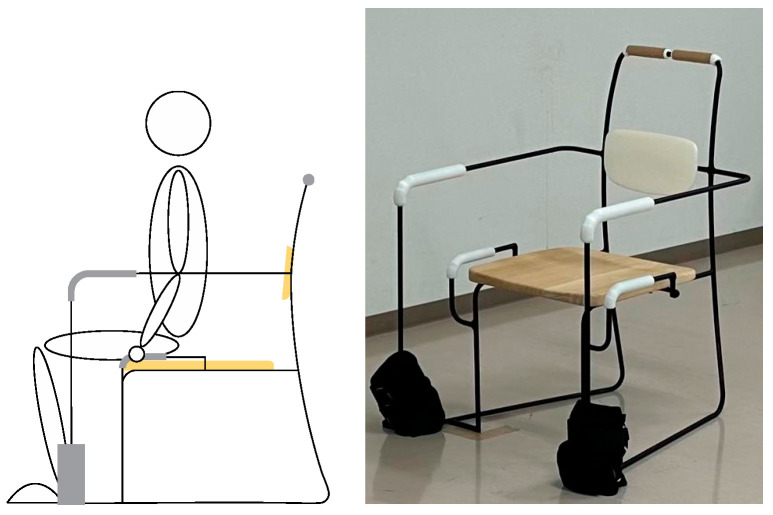
The figure shows the use of a modified chair while performing seated resistance training.

The grips are set at the same height as the seating surface, 7 cm from the seat’s base. This setting allows the elderly to use the grips in a natural posture, which reduces strain on the body and provides stable support (Figure 1). Furthermore, the diameter of the grips is ergonomically designed for ease of gripping and to minimize hand fatigue [28].

#### 2.2.2. Problems 2 and 3

**Figure 2:**
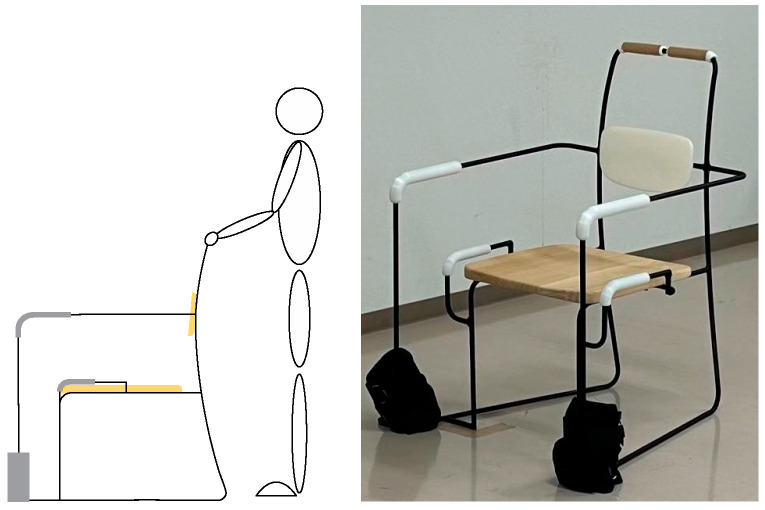
The figure shows the use of a modified chair while performing standing resistance training.

The solution to problems 2 and 3 in this study was to redesign the chair’s backrest. Developed based on the average height and the natural reachable position of a person when standing, the chair has a maximum height of 90 cm [28]. This allows the elderly person to easily maintain body stability during resistance training while preventing the center of gravity from shifting forward. In addition, a removable 10-kg weight is placed on the front of the chair. These weights improve overall stability and prevent the chair from tipping backward during resistance training due to the force from the elderly person’s movements. This further enhances safety during exercise and provides a safe environment for the elderly person to focus on their resistance training (Figure 2). These improvements are expected to significantly increase the safety and effectiveness of resistance training for the elderly.

#### 2.2.3. Problem 4

**Figure 3:**
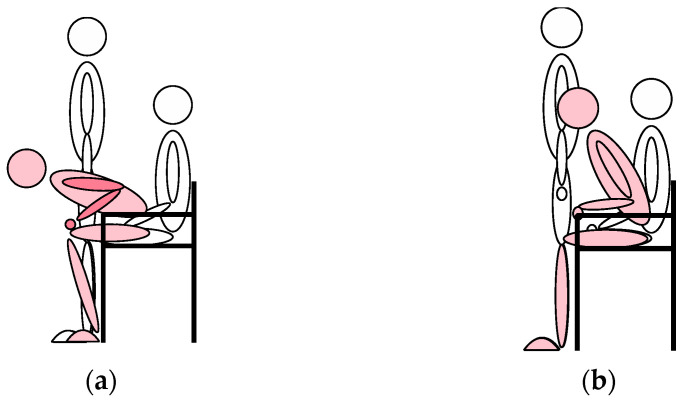
The figure shows the process of standing up in the correct way (**a**) and an inadequate center of gravity transfer to stand up (**b**).

According to previous research [31], the correct process of standing up from a chair is to lean the body forward to shift the center of gravity and then stand up using primarily the strength of the lower extremities (Figure 3a). In this posture, the risk of falling is reduced, and one can exert one’s strength more efficiently. In an incorrect standing posture, there is often an inadequate center of gravity transfer and excessive use of the hands and arms (Figure 3b). This makes it easier to lose balance and increases the risk of falls.

**Figure 4:**
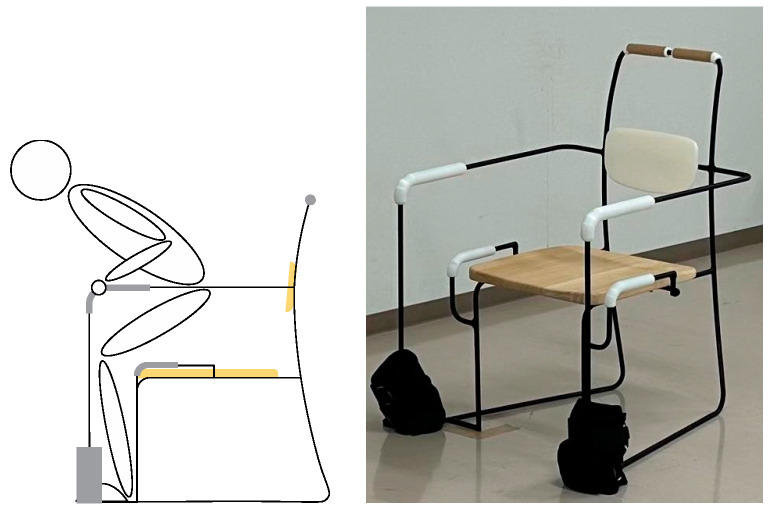
The figure shows the use of a modified chair while performing stand-up resistance training.

The chair’s armrests were extended 15 cm forward and 15 cm to the left and right, integrating the armrests with the entire chair to assist the elderly person in training to stand up correctly and maintain a sufficient range of activity [28]. This design allows the elderly person to train standing up while maintaining the correct posture (Figure 4), and for frail elderly individuals, it also serves to maintain body stability.

### 2.3. Experiment

A specific experiment was conducted to quantitatively verify how the developed chair described in the previous section affects the physical stability of older adults during resistance training. The primary objective of this experiment was to scientifically evaluate whether the proposed chair would enhance safety and comfort during exercise for the elderly. The effectiveness of the developed chairs in assisting older adults to maintain balance and properly move during resistance training was compared to regular chairs.

**Figure 5:**
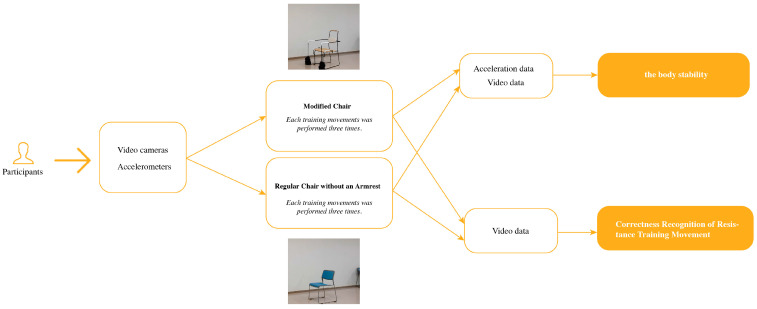
This figure shows the research process.

In this experiment, 19 older adults were asked to perform a series of designated exercises in the developed chair and a chair without armrests. Each participant was fitted with an accelerometer to record their body movements during the exercise, and three additional video cameras were used to capture the movements from different angles. This allowed us to collect data on their movements during exercise, particularly for balance and stability. The collected data will later be used as the basis for a detailed statistical analysis to evaluate the specific effects of the modified chair on the physical stability of the elderly. Additionally, by analyzing the video data, we will calculate the accuracy of the elderly participants’ movements. This analysis will be used to develop a model for the correctness recognition method of resistance training movement, aimed at improving the correctness of the movements performed. Figure 5 shows the general process, from how data are collected to how comparative experiments are conducted and how data are analyzed.

### 2.4. Participants

Nineteen older adults (age: 61–80) participated in this experiment, with an average age of 72.1 (SD 4.7). The group comprised 11 men and 8 women. All the participants were briefed on the purpose and procedure of the experiment and agreed to participate. The selected participants were older adults aged 60 and above who were in a healthy state, capable of living independently, and able to perform simple physical exercise routines. There was no requirement for the participants to have a regular exercise habit. Additionally, there were no restrictions regarding conditions such as leg pain, to ensure the data would be representative of a wide range of older adults.

### 2.5. Experimental Environment and Tools

**Figure 6:**
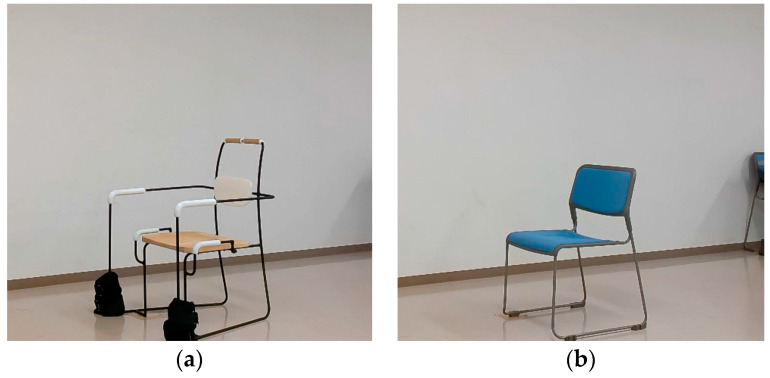
This figure shows a modified chair (**a**) and a regular chair without an armrest (**b**).

The experiment took place in a controlled environment, where participants performed specified resistance training in both the modified chair and a regular chair (CK-S890VR64N) (Figure 6). To record body movements in detail, the participants wore accelerometers while training.

#### 2.5.1. Accelerometer Model

In this study, the WT901BLECL5.0 model (China) triaxial accelerometer was utilized. This device is capable of measuring acceleration, angular velocity, and quaternion data. The accelerometer was positioned on the chest of the participants, approximately at the level of the T12 vertebra. This placement is beneficial for accurately capturing movements of the upper body core and is commonly used in research analyzing centers of mass and other related metrics [32].

#### 2.5.2. Video Cameras

Three video cameras were used to simultaneously record images from different angles to analyze the participant’s movements during the exercise. The camera employed in this study was configured to capture video data at a resolution of 1080P and a frame rate of 30 frames per second. To more clearly record the participants’ motion information, cameras were positioned directly in front of and behind the participants, as well as at a 45-degree angle above the left side.

### 2.6. Measurement Method and Data Analysis

The collected acceleration data were analyzed to measure the stability of the participants’ movements. Specifically, the variation in acceleration during resistance training was analyzed to compare the difference in stability between the developed chair and the chair without armrests.

It was necessary to convert the sensor data to an earth coordinate system to use the accelerometer data collected in the experiment for an actual motion analysis. A rotational transformation using quaternions was used, based on the following formula:

*a*_e_ = *q* · *a*_s_ · *q*∗,

*a*_e_ is the acceleration vector in the earth coordinate system; *a*_s_ is the acceleration vector in the sensor coordinate system; *q* is a quaternion representing the rotation from the sensor to the earth coordinate system; and *q*∗ is the conjugate quaternion of the quaternion *q*.

Transformation by quaternions allows accurate orientation from any sensor rotation to the earth coordinate system and corrects for the direction of acceleration. Since the primitive data recorded by the sensor depend on its orientation, acceleration data consistent with the actual direction of motion can be obtained through this transformation.

To quantitatively evaluate the effect of the developed chair on the physical stability of an elderly person during resistance training, we calculated the composite acceleration in the horizontal (XY) direction and its root mean square (RMS) value. This differs from the composite acceleration in the XYZ direction, which is the conventional method for evaluating body stability [33,34]. Previous studies have shown that the RMS value of the composite acceleration in the XY direction effectively evaluates body stability [35]. The use of the RMS values of the horizontal composite acceleration to compare the modified chair with the ordinary chair shows that both are equally effective in maintaining body stability. This study excluded data in the Z direction because it includes exercises with significant vertical (Z-direction) acceleration fluctuations, especially in standing training. This is because vertical acceleration variations are due to individual differences in the physical fitness of the elderly, which may have a non-uniform effect on the experimental data.

Therefore, this study employed this indicator to analyze whether the developed chair contributed to improved stability during exercise. Specifically, the RMS values of the composite acceleration in the XY direction were measured before and after the exercise performed by the participants using both chairs, and we compared the results from both.

### 2.7. Statistical Procedures

In this experiment, the nineteen older adult participants were not divided into separate groups. The primary aim was to obtain data representative of a broad range of older adults without specific conditions or habits.

All the raw data were inspected for completeness and accuracy. To enhance the analysis, data segments captured during periods when participants were stationary, as identified from video recordings used in the experiment, were excluded.

The acceleration data for each action performed by each participant were measured using both modified chairs and standard chairs without armrests. The root mean square (RMS) values of these acceleration data were then calculated to obtain the average RMS values for each action. The data were subsequently categorized based on the type of chair used and the specific actions performed.

Alongside the physical measurements, a detailed questionnaire survey was conducted with 15 of the 19 participants. The questionnaire utilized a 7-point scale, ranging from 1 to 7, where participants rated each question, with higher scores indicating better evaluations. This survey involved calculating the average scores for each action using each type of chair, focusing on body stability during the training and the comfort of use.

Finally, a comprehensive analysis was conducted to determine whether the modified chair design could improve the body stability of older adults.

### 2.8. Correctness Recognition Method of Resistance Training Movement

#### 2.8.1. MediaPipe

MediaPipe is a machine learning framework developed by Google, specializing in real-time media processing. It is widely used for processing video, image, and audio data and provides advanced vision models for face recognition, hand tracking, and posture estimation, among other applications. It has been widely adopted by researchers and developers. For example, a study on yoga posture recognition proposed a novel architecture that utilizes MediaPipe to skeletonize yoga poses from images and classify them with high accuracy [36]. Other studies have employed similar principles, initially identifying key points on the body and subsequently determining postures based on these images [37].

In contrast to methods requiring expensive hardware, such as those using the Kinect sensor, this study employs MediaPipe technology, which leverages a standard computer, smartphone, or tablet camera to capture movement [38]. This approach is cost-effective, simple to operate, and particularly suitable for elderly individuals to self-manage and monitor the quality of their exercise over time at home.

#### 2.8.2. Method of Deriving Joint Angles

**Figure 7:**
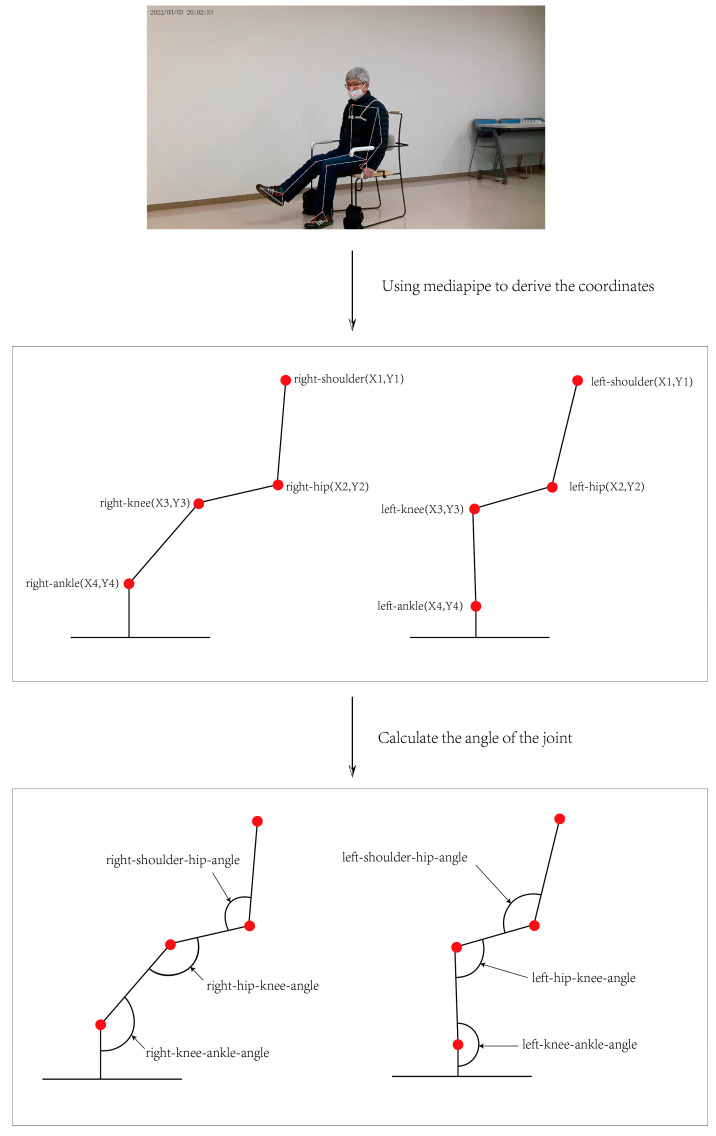
The figure shows how joint angle data can be extracted from a video.

In this study, the process of deriving joint angles begins with the capture of images. The video data used were from the camera positioned at a 45-degree angle above the participant’s left side. MediaPipe technology is utilized to automatically recognize critical human body parts within these images and output their two-dimensional coordinates. MediaPipe employs advanced machine learning models to recognize human poses in real-time, providing outputs that include the coordinates of joint points such as shoulders, hips, knees, and ankles. The process of extracting coordinate points as well as angles from the image is shown in Figure 7.

Using these coordinate points, we calculated the angles of each joint using vector analysis. The specific calculation process involves first defining vectors between adjacent joint points, then calculating the angle between two vectors using the dot product formula, thereby determining the angles of the joints. The formula for calculating the angles is as follows:

$$\theta =\cos^{-1}\left(\frac{{\vec{\nu }}_{1}\cdot {\vec{\nu }}_{2}}{\left|{\vec{\nu }}_{1}\right|\times \left|{\vec{\nu }}_{2}\right|}\right),$$

The vectors ${\vec{\nu }}_{1}$ and ${\vec{\nu }}_{2}$ represent two joint vectors, and *θ* is the angle between these two vectors.

#### 2.8.3. Data Windowing and Feature Extraction

This study adopted windowing processing to effectively utilize joint angle data. Specifically, each window consisted of 30 data points, with an overlap of 15 data points between consecutive windows. This setup minimizes the loss of information while maintaining the continuity of the data.

For the windowed dataset, the following statistical characteristics were calculated to evaluate the dynamic changes in joint angles:

Mean: The average of the data points in each window, reflecting the general trend of the joint angles during the motion cycle.

Minimum and Maximum Values: The minimum and maximum values from the data points within each window identify the extreme peaks and valleys during motion.

Median: The median of the data in each window, which identifies the central tendency less influenced by outliers.

Standard Deviation: Represents the variation in data, assessing the consistency and magnitude of variations in motion.

Furthermore, to explore the characteristics in the frequency domain, power spectral analysis was also performed. The specific methods are as follows:

Power Spectrum Calculation: The Welch method was applied to the data in each window to calculate its power spectrum.

Power Spectrum Statistics: Within a specified frequency range (1–20 Hz), the mean, maximum, minimum, median, and standard deviation of the power spectrum were calculated.

The results of these statistical measures and power spectral analysis are recorded and stored as features for motion recognition.

#### 2.8.4. Principal Component Analysis (PCA)

This study used principal component analysis (PCA) to analyze joint angle data. The primary purpose of this analysis is to extract key features that effectively distinguish between accurate and inaccurate movements from complex motion data. Initially, PCA was performed separately for each movement using 60 feature variables. However, it was found that when all 60 feature variables were analyzed together, the PCA plots did not show significant differences between accurate and inaccurate movements. Therefore, the researcher proceeded to screen the 60 feature variables, ultimately identifying the most characteristic features for each movement. PCA performed on each movement allowed for the identification of principal components directly related to the accuracy of the movements. These principal components were used as input features in a machine learning model, aimed at enhancing the model’s recognition ability.

#### 2.8.5. Classification Using Support Vector Machines (SVMs)

This study used support vector machines (SVMs) to classify each movement as correct or incorrect using features extracted through principal component analysis (PCA). Table 2 shows the selected features for each movement. This approach is intended for the automatic identification and evaluation of movement accuracy. Individual SVM models are trained using the most distinct features identified by PCA for each movement.

The movement dataset is constructed based on features selected from prior research. Leave-one-out cross-validation (LOOCV) is conducted for each movement. This method implies that each sample is used once as test data, while the model is trained on all other samples. This allows for the maximum assessment of the model’s generalizability and robustness.

## 3. Results

### 3.1. Results of Modified Chair

**Figure 8:**
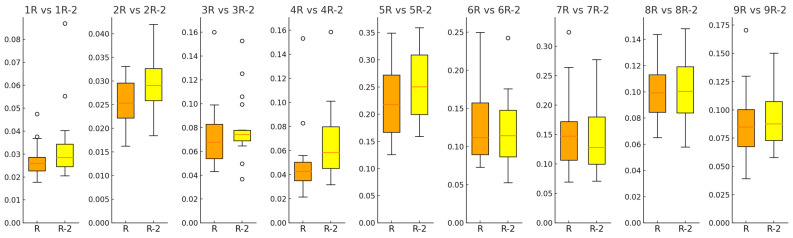
The figure shows the statistical results of the acceleration RMS values.

In this study, the RMS values of the composite acceleration in the XY direction during the exercises were calculated and visually displayed in box plots (Figure 8). Significant differences were observed between the modified chairs (labeled R) and regular chair without an armrest (labeled R-2), particularly in sitting movements (1R to 4R, 1R) and initial standing movements (5R), where the numbers before R and R-2 represent the movement numbers. The box plots of the modified chairs show a lower median and narrower interquartile range in these movements, suggesting greater stability. However, no significant differences were observed in extended-standing movement exercises (6R to 9R).

The statistical analysis of the RMS values revealed that the chair under development significantly improved body stability in seated resistance training compared to the conventional chair (*p* < 0.001). A similar significant difference was observed in standing resistance training (*p* < 0.001), indicating that the developed chair provides effective stability during these movements.

**Figure 9:**
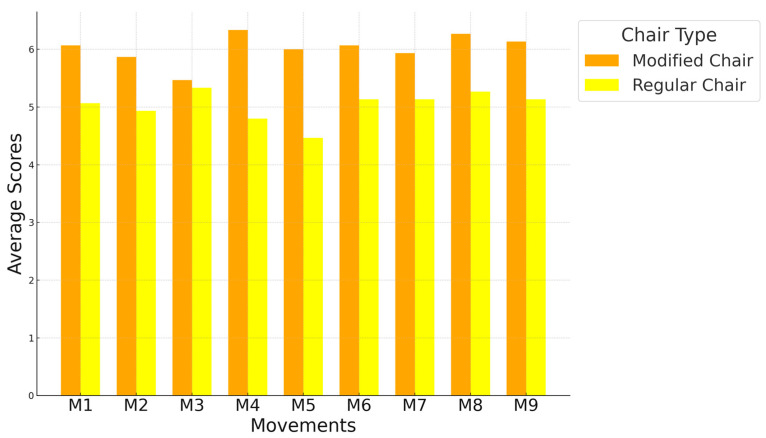
The figure shows the average scores for each movement using each type of chair, based on body stability during the training.

**Figure 10:**
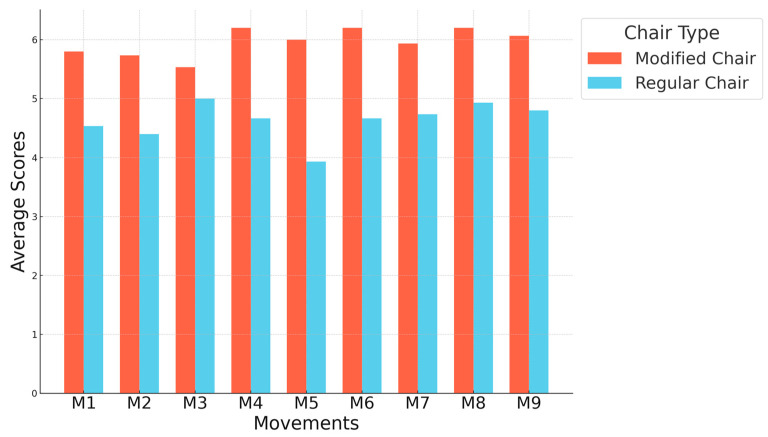
The figure shows the average scores for each movement using each type of chair, based on the comfort of use.

For the study, 15 out of 19 participants completed two identical questionnaires documenting their experiences with two different chairs. The experiment consisted of nine movements, with each movement associated with two questions that were consistent across all movements. The first question assessed body stability during the training, and the second question evaluated the comfort of use. Figure 9 and Figure 10 show that for each training movement, the modified chair received significantly higher ratings than the regular chair without an armrest.

### 3.2. Correctness of Movement

Video data collected during the experiment revealed a critical issue: despite the assistance of the improved chair, the participants’ movement accuracy rate, the individuals’ ability to properly conduct the exercise, was only 59.75% during movement activities besides standing resistance training (Table 3). The data for the correct and incorrect movement are shown in Table 3.

### 3.3. The Results of the PCA Analysis

**Figure 11:**
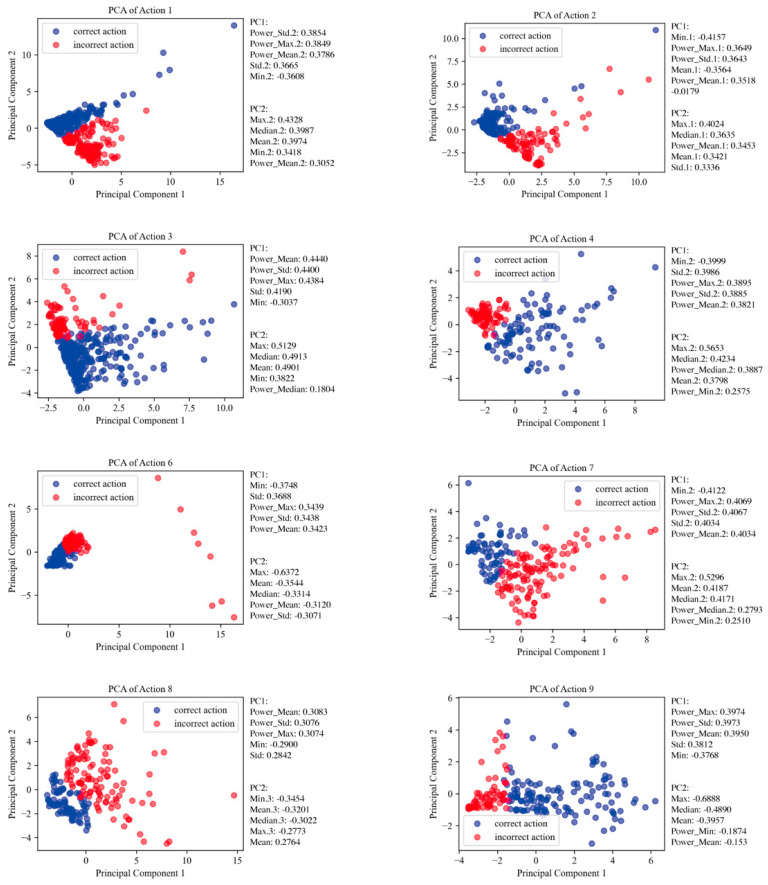
This figure displays a PCA analysis aimed at extracting key features that effectively differentiate between accurate and inaccurate movements from complex motion data. For the left side, the data include left shoulder-hip angle metrics such as Mean, Min, Max, Std, Median, and Power Spectrum Statistics; similar metrics for the left hip-knee angle are denoted as Mean.1 through Power_Median.1; left knee-ankle angle data extend from Mean.2 through Power_Median.2. On the right side, the statistics shown are for the right shoulder-hip angle (Mean.3 through Power_Median.3), right hip-knee angle (Mean.4 through Power_Median.4), and right knee-ankle angle (Mean.5 through Power_Median.5).

Graphs of the PCA analysis for eight movements were created; these graphs demonstrate the contribution of the selected features in distinguishing between accurate and inaccurate movements, thereby proving the efficacy of the features. The results of the PCA analysis specific to each action are shown in Figure 11.

### 3.4. Results of SVM Model

Below, an integrated graph of the performance evaluation is presented, including the average accuracy of the SVM models for the eight movements. This graph provides a detailed depiction of the performance of the SVM models based on the PCA features for each movement, showing the accuracy, precision, recall, and F1 score for each class. This demonstrates the overall effectiveness of the models and assesses the likelihood of recognizing each separate movement. Data such as the correctness of each movement model are shown in Table 4.

## 4. Discussion

The modified chair was found to be effective in maintaining physical stability over conventional chairs in elderly individuals during sit-to-stand movements and seated resistance training. On the other hand, no statistically significant differences were observed in the movements performed in a standing posture. This may be because the supportive action of the chair on the body was not as pronounced when performing resistance training in a standing position, and thus, no clear differences were observed. Additionally, the participants were elderly people in good physical condition, which may also contribute to the lack of significant differences. Furthermore, when using a regular chair, it was observed that the chair’s low height caused the elderly to lean forward during resistance training, shifting the body’s center of gravity forward.

Video data confirmed that accurate sit-to-stand movements were maintained, allowing participants to complete the training correctly. However, the accuracy rate for other resistance training movements using the modified chair remained at 59.75%. This finding suggests that enhancements in physical support systems alone are insufficient to fully address the problem of movement accuracy. Consequently, there is an urgent need to develop systems capable of identifying and instructing resistance training movements in real time to increase resistance training effectiveness, reduce error rates, and thereby decrease the risk of potential injuries.

In this study, the movement correctness was determined using data on changes in joint angles during exercise. Through a PCA analysis and SVM model training, it was demonstrated that this method could accurately recognize the correct movements. Changes in joint angle data are less affected by variations in camera angles compared to positional data of keypoints used in other posture classification-related studies [39]. However, this paper does not specifically quantify how much these influences are reduced compared to different angles.

Although the elderly rate the user experience of the modified chair higher than regular chairs, this improvement potentially alleviates some issues with their lack of enthusiasm for exercise. However, the current system still lacks sufficient support to help elderly individuals continue resistance training in the long term and enhance their enthusiasm for such training. This situation necessitates comprehensive improvements in the system. Moreover, the current system can only recognize the correct movements and is not yet capable of identifying different types of movements. Therefore, classifying and analyzing movements in detail remains an important topic for future research.

## 5. Limitation

### 5.1. Number of Participants and Health Status

This study involved 19 elderly participants. The small sample size limits the generalizability of the results. All the participants were relatively healthy elderly individuals. Future research should further investigate the applicability of these results to elderly populations with diverse health conditions, to enhance the reliability and generalizability of the research.

### 5.2. Diversity of Resistance Training

The modified chair used in this study showed effectiveness in specific resistance training, but further verification is needed to determine its effectiveness for other types of resistance training. Future studies should continuously adjust the chair’s design to accommodate different forms of resistance training, further enhancing its effectiveness.

### 5.3. Limitations of the SVM Model

When assessing the accuracy of movements using MediaPipe technology, the background color and lighting conditions may lead to inaccuracies in joint angle calculations, impacting the analysis and its results. Future research should reanalyze and improve the environmental conditions and refine the algorithm to minimize the impact of these external factors. Moreover, the study does not conduct experiments to assess the influence of video angles on accuracy.

## 6. Conclusions

In this study, the modified chair significantly improved body stability in seated resistance training compared to the conventional chair (*p* < 0.001). A similar significant difference was observed in standing resistance training (*p* < 0.001), indicating that the developed chair provides effective stability during these movements. The results of the questionnaire survey indicate that older adults rated the modified chair higher for all movements compared to the standard chair. Compared to the regular chair without an armrest, the modified chair provided a greater sense of security and a better user experience for older adults. These findings contribute to the body of knowledge on specialized equipment designed specifically for training that can reduce risk and enhance engagement in physical activities among elderly populations, underscoring the potential for design innovation to improve the quality of life and promote healthier aging through better support and safety in exercise routines.

This study utilizes data on changes in joint angles to determine the correctness of movements, achieving a high accuracy rate in distinguishing between correct and incorrect executions of nine specific movements (mean 97.68%). It provides a research direction for subsequent researchers conducting studies on resistance training for the elderly, offering a reliable method for assessing and improving the precision of exercise techniques to enhance safety and efficacy in geriatric fitness programs. Additionally, the assessment can be easily conducted by capturing video data through ordinary computer or smartphone cameras. The application of this method has significantly optimized the posture recognition process, making training guidance more scientific and precise. Continuous monitoring of joint angles allows for the immediate detection and correction of incorrect postures, preventing potential injuries during resistance training and enhancing researchers’ understanding of how different parts of the body coordinate during physical activities for the elderly.

Future research directions include integrating other types of assistive devices (such as smart wearable devices) with the developed chair to enhance the safety and effectiveness of training further [40]. And improvements in the safety of the armrest position and height dimensions of the chair will be made to adapt to more people of different heights. The aim is to optimize motion recognition algorithms and combine them with other measurement data, such as electromyography, to improve the accuracy and response speed of motion recognition. Longitudinal studies on elderly individuals will be conducted to assess the impact of continued use of the improved chair and motion recognition system on elderly health.

It is crucial for elderly individuals to achieve the correct body height and maintain appropriate joint angles throughout the exercises. However, some elderly individuals with weaker physical strength may find it difficult to meet these standards in the initial stages. Therefore, designing different exercises of varying difficulty suitable for elderly individuals with varying health conditions is an important direction for future research. This will allow for the development of more personalized and effective exercise plans adapted to the diversity and dynamic changes in elderly individuals’ physical capabilities.

## Figures and Tables

**Table 1:** Summary of the Problems Identified with Using Armless Chairs during Resistance Training.

Areas of Concern		Problem Number	Details
Resistance training performed in a seated position	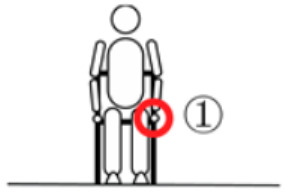	1	The chair does not provide a space where the hands can be placed on both sides. The elderly individuals maintain physical stability during training exercises by gripping the seat. However, as they age, their grip strength diminishes, which can lead to an inability to hold onto the seat effectively [27]. This makes it difficult for the elderly to properly support their bodies when performing exercises. This may also cause a shift in the elderly person’s body’s center of gravity.
Resistance training performed in a standing position	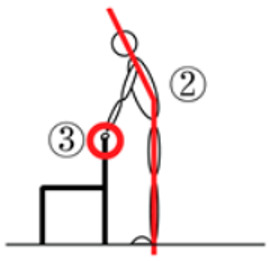	2	The height of the chair’s backrest is not appropriate. The elderly may have difficulty standing completely upright when trying to maintain equilibrium during exercises, which may increase the risk of injury from falls [28].
		3	The chair’s backrest does not provide an area for the hands. When maintaining equilibrium, the hands cannot firmly hold the chair in place, which could increase the risk of falls [29].
Stand-up resistance training	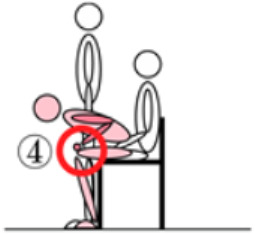	4	During stand-up training, current chairs lack a design that accommodates the maintenance of the elderly person’s center of gravity. The process of standing up requires a forward shift of a person’s center of gravity before standing. However, the armrests of the chairs are not suitable for this type of training for physically weak elderly individuals [30].

**Table 2:** The Table Shows Selected Features for Each Movement.

Movement	Selected Features
M1	left knee-ankle angle data
M2	left hip-knee angle data
M3	left shoulder-hip angle data
M4	left knee-ankle angle data
M6	left shoulder-hip angle data
M7	left knee-ankle angle data
M8	left shoulder-hip angle data and right shoulder-hip angle data
M9	left shoulder-hip angle data

**Table 3:** The Table Presents the Counts of Correct and Incorrect Detections for Each Movement Type During the Experiment.

Movement	M1	M2	M3	M4	M6	M7	M8	M9	Total
Correct	202	167	286	100	156	72	82	130	1195
Error	146	103	100	75	89	119	109	64	805

**Table 4:** The Table Shows the Data Related to the Correctness of Each Movement Model.

Average Accuracy 1: 98.8506%				
	Precision	Recall	F1-score	Support
0	0.97	1	0.99	146
1	1	0.98	0.99	202
Accuracy			0.99	348
Macro Avg:	0.99	0.99	0.99	348
Weighted Avg:	0.99	0.99	0.99	348
Average Accuracy 2: 99.6296%				
	Precision	Recall	F1-score	Support
0	0.99	1	1	103
1	1	0.99	1	167
Accuracy			1	270
Macro Avg:	1	1	1	270
Weighted Avg:	1	1	1	270
Average Accuracy 3: 97.9275%				
	Precision	Recall	F1-score	Support
0	0.94	1	0.97	100
1	1	0.98	0.99	286
Accuracy			0.98	386
Macro Avg:	0.97	0.99	0.98	386
Weighted Avg:	0.99	0.98	0.98	386
Average Accuracy 4: 99.4286%				
	Precision	Recall	F1-score	Support
0	0.99	1	0.99	75
1	1	0.99	0.99	100
Accuracy			0.99	175
Macro Avg:	0.99	0.99	0.99	175
Weighted Avg:	0.99	0.99	0.99	175
Average Accuracy 6: 95.5102%				
	Precision	Recall	F1-score	Support
0	0.89	1	0.94	89
1	1	0.93	0.96	156
Accuracy			0.96	245
Macro Avg:	0.95	0.96	0.95	245
Weighted Avg:	0.96	0.96	0.96	245
Average Accuracy 7: 97.9058%				
	Precision	Recall	F1-score	Support
0	0.99	0.97	0.98	119
1	0.96	0.99	0.97	72
Accuracy			0.98	191
Macro Avg:	0.98	0.98	0.98	191
Weighted Avg:	0.98	0.98	0.98	191
Average Accuracy 8: 95.2880%				
	Precision	Recall	F1-score	Support
0	1	0.93	0.96	109
1	0.91	1	0.95	82
Accuracy			0.96	191
Macro Avg:	0.96	0.96	0.96	191
Weighted Avg:	0.96	0.96	0.96	191
Average Accuracy 9: 96.9072%				
	Precision	Recall	F1-score	Support
0	0.94	1	0.97	64
1	1	0.97	0.98	130
Accuracy			0.98	194
Macro Avg:	0.97	0.98	0.98	194
Weighted Avg:	0.98	0.98	0.98	194

In the table, 0 and 1 represent the labels for incorrect and correct movements, respectively.

## Data Availability

The authors will make the raw data supporting this article’s conclusions available upon request.
